# Infantile Colic: When to Suspect Cow’s Milk Allergy

**DOI:** 10.3390/nu17223600

**Published:** 2025-11-18

**Authors:** Yvan Vandenplas, Silvia Salvatore, Mario C. Vieira, Francesco Savino, Ralf G. Heine, Koen Huysentruyt, Rosan Meyer

**Affiliations:** 1 KidZ Health Castle, UZ Brussel, Vrije Universiteit Brussel, 1090 Brussels, Belgium; koen.huysentruyt@uzbrussel.be; 2 Pediatric Unit, Hospital “F. Del Ponte”, Department of Medicine and Technological Innovation, University of Insubria, 21100 Varese, Italy; silvias.varese@gmail.com; 3 Centro de Gastroenterologia Pediátrica, Hospital Pequeno Príncipe, Curitiba 80250-060, Brazil; vieira.mcv@gmail.com; 4 “Regina Margherita” Children Hospital, Department of Pediatric, University of Torino, 10126 Torino, Italy; francesco.savino@unito.it; 5 Population Health, Murdoch Children’s Research Institute, Parkville, Melbourne 3052, Australia; ralf.heine@mcri.edu.au; 6 Department of Medicine, KU Leuven, 3000 Leuven, Belgium; rosan.research@rosan-paediatricdietitian.com

**Keywords:** infantile colic, cow’s milk allergy, infant distress, crying

## Abstract

**Background/Objectives:** Worldwide, an estimated 20–30% of infants suffer from infant colic (IC), with excessive crying and unsettled behavior, during the first three months of life. These infants are often referred for a medical evaluation, but the pathogenesis of IC remains poorly understood. The aim of this narrative review is to critically appraise the available literature regarding the relation between IC and cow’s milk allergy (CMA). **Methods:** A literature search using the search strings cow’s milk allergy [MeSH Terms] OR food allergy [MesH Terms] AND colic [MeSH Terms] OR crying [MeSH Terms], limited to the English language, from inception to 15 June 2025, resulted in the identification of 135 articles. Of these, 18 clinical trials assessed the effect of a cow’s milk elimination diet on IC. **Results:** The role of CMA in IC in the absence of other allergic manifestations remains uncertain. However, when standard treatment of infant colic has failed and when other allergic symptoms are present, CMA may be considered. A diagnostic elimination diet which includes a 2–4-week trial of maternal cow’s milk elimination in breastfed infants or an extensively hydrolyzed cow’s milk or hydrolyzed rice formula should be performed. If the elimination diet results in a significant decrease in symptoms, reintroduction of cow’s milk protein into the diet is mandatory to fulfill the diagnostic criteria of CMA. **Conclusions:** Considering the limited current evidence, future research should prioritize large well-designed clinical trials with a focus on investigating CMA in colicky breastfed and formula-fed infants.

## 1. Introduction

Worldwide, an estimated 20–30% of infants suffer from infantile colic, a pattern of excessive crying with unknown cause that typically starts within the first few weeks of life and improves without intervention towards 4–5 months of age [1,2,3]. Crying is part of normal infant behavior and a way of communication, usually to express hunger, tiredness, discomfort, or a request for comforting stimulation [4]. However, some infants cry louder and longer than others for no apparent reason, particularly in the afternoon or early evening during the first months of life. When an infant cries inconsolably or when this is judged to be excessive by caregivers, it can lead to significant distress, may disrupt parenting and, in rare cases, may place an infant at risk for physical abuse [5]. Tolerance for a crying baby varies among parents but has in general reduced over time [5]. There are no data on racial differences in the prevalence of infant colic, but the parental tolerance to what is considered normal crying may depend on the socio-economic background [5]. Adult brains have evolved to become hypersensitive to infant cries, and babies respond to parental stress by crying more, thus setting up a vicious cycle [6]. The psychosocial implications of infantile colic and the placebo effect in the management of infant colic cannot be neglected [6], and it is important for healthcare professionals to focus on reducing parental anxiety by offering reassurance and support [6].

There is no agreement on what exactly constitutes excessive crying. Initially, Wessel et al. established in 1954 diagnostic criteria for infantile colic, defining it as a condition in which otherwise healthy and well-nourished infants exhibit episodes of unexplained crying lasting more than three hours per day, on more than three days per week, for a minimum duration of three consecutive weeks [7]. In 1962, Brazelton analyzed the diaries of 80 mothers attending a private pediatric practice in Massachusetts, USA, and asked to record their infants’ daily cry durations over the first 12 weeks of life [4]. There was an average of 2¼ hours of crying in the first 7 weeks, with a gradual reduction each week thereafter [4]. A meta-analysis pooling parent-reported data from 7580 infants aged up to 12 months, from 17 countries and 57 studies, confirmed a crying peak at 5–6 weeks of age and a pooled estimate for daily cry and fuss duration of 130 ± 61 min, with high heterogeneity [8]. The determination of crying time as part of normal behavior in presumed healthy infants varies between countries [1,5].

**Figure 1:**
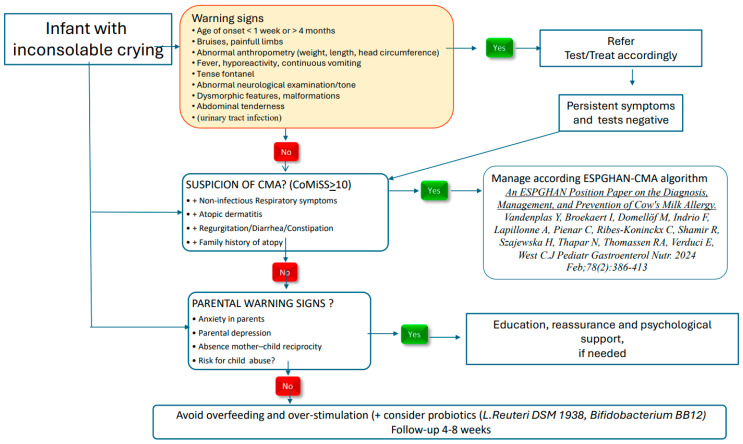
Recommended management in infantile colic [14].

Infants presenting with crying in association with concerning symptoms or underlying health issues (i.e., fever, lethargy, abnormal psychomotor development, seizures, vomiting, dehydration, faltering growth), acute or chronic, are beyond the scope of this manuscript and should be carefully evaluated and monitored by healthcare professionals (HCPs) (Figure 1). However, the published literature on colic does not always clearly separate crying in presumed healthy infants from that occurring in infants with an underlying disease or condition.

Cow’s milk allergy (CMA) is one of the most common food allergies in infants, with an estimated prevalence ranging from 1.8% to 7.5%, as reported between 1973 and 2008 [9]. In 2015, the EuroPrevall birth cohort study based on 12,000 infants found an overall incidence of 0.54% for both IgE- and non-IgE mediated CMA, as confirmed by oral food challenge [10]. The European Academy of Allergy and Clinical Immunology (EAACI) has published three position papers on complex and controversial topics in non-IgE-mediated allergy: the diagnosis and management of non-IgE-mediated food allergies in breastfed infants, food protein-associated gastroesophageal reflux disease (GERD) and constipation [11,12,13].

The controversy of colic as a symptom of CMA was briefly covered in the position paper on non-IgE-mediated allergies in breastfed infants [11]. However, infant crying and/or colic warrants a full review of the literature with guidance on all infants, irrespective of the mode of feeding. Because there is still debate about the relation between infant colic and CMA, we reviewed literature on “infant colic and CMA”. This review aims to clarify when to consider CMA in infants in whom recommended management of infant colic fails. Practical guidance to healthcare professionals (HCPs) is proposed when CMA is considered as a possible etiology of infant colic [14].

## 2. Literature Search Strategy and Results

According to the purpose of this review, the literature was searched (PubMed^®^, Embase, Cochrane database) using the search string Cow’s milk allergy [MeSH Terms] OR Food allergy [MesH Terms] AND colic [MeSH Terms] OR Crying [MeSH Terms], limited to the English language, from inception to 15 June 2025. One hundred and thirty-five articles were identified, including case reports, observational and intervention studies, guidelines and reviews. Collection of references from already included papers also occurred to retrieve additional studies. Eighteen clinical trials assessed the effect of a cow’s milk elimination diet in infantile colic and are summarized in this review (Table 1). Since we did not adopt the methodology for a formal systematic review, we cannot exclude that other relevant studies on this topic may have been missed. Infant colic is typically considered as a non-IgE-mediated manifestation of CMA [14]. Testing for IgE-mediated allergy was performed in one trial, showing a positive SPT in 3/114 infants presenting with colic (Table 1).

To improve clinical practice and the implementation of this review, the authors provided statements and agreement. Statements were developed via correspondence based on the collective insights of the authors. All co-authors voted anonymously online. Each participant had the option to either agree or disagree with the statements (voting between 0 and 9, with 6 and above meaning agreement, and 5 and below meaning disagreement). A predefined agreement threshold of 70% (≥5/7 authors) was set to determine consensus—statements receiving less than 70% agreement were considered to have significant disagreement.

## 3. Definition of Infantile Colic

The term colic is derived from “kolikos”, the Greek word for colon. Historically, colonic gas and pain were commonly used to explain colic in a young infant. Infantile colic is considered a self-limiting Disorder of Gut–Brain Interaction (DGBI), formerly referred to as Functional Gastrointestinal Disorder (FGID) [3,31]. Typical colic symptoms include excessive crying, leg flexion, back arching, limb stiffening, flatulence and abdominal distension.

According to the current Rome IV diagnostic criteria for DGBI, a diagnosis of infant colic is made based on the presence of recurrent periods of inexplicable crying, fussing or irritability in an apparently healthy infant aged less than 5 months [3]. The 2016 Rome IV criteria no longer mention “3 h per day” as a cut-off duration because this time window was considered as arbitrary and not focused on the prolonged, inconsolable characteristic of the crying episodes which may trigger caregiver distress [3]. Moreover, the term ‘paroxysmal’ present in the Rome III criteria was abandoned, since evidence is lacking to support that infant colic differs in sound or starts more abruptly compared to normal crying bouts [3].

## 4. Etiology of Infantile Colic

The etiology of colic is not known, and multiple hypotheses have been proposed, including disturbances of gastrointestinal motility, visceral hyperalgesia, gut microbiota, hormones, feeding technique, diet, infant–parent interaction, parental stress and coping, neurobehavioral development, and early life events [32]. It appears likely that each of the forementioned hypotheses accounts for the inconsolable crying in a proportion of the population of infants with colic.

The different etiologies of colic can be classified as non-gastrointestinal or gastrointestinal. The former include poor feeding techniques, modified child–parent relations, immaturity of the central nervous system, behavioral conditions, and environmental factors such as maternal smoking or nicotine replacement therapy [2].

Infant colic is classified as a disorder of DGBI, although there are many other reasons for infants to present with excessive crying. Clinical trials have shown that the majority of infants do not present with an isolated DGBI, but crying is often part of a combination of different manifestations or symptoms of DGBI [33,34]. Colic associated with regurgitation has been attributed to GERD [35]. While the “happy spitter” does exist, the majority of infants that frequently regurgitate are also often distressed and cry [36]. The chemical content of refluxate may also have a different effect on symptoms, and non-acid reflux has been more closely associated with infant distress than acid reflux [37], likely related to the stimulation of volume receptors in the esophagus. Nevertheless, there is no proven causal relationship between crying duration and the severity of acid reflux, as measured by esophageal pH monitoring [38,39]. However, the assumed association between colic and reflux disease has led to an overtreatment of infants with acid-suppressive mediations, despite clinical trials showing that these medications do not provide any symptomatic benefit for excessive crying [38].

Alternatively, the gastrointestinal causes may involve immunoglobulin (Ig)E- and non-IgE-mediated food allergy (in particular, CMA), gut microbial dysbiosis or gastrointestinal inflammation that may cause intestinal hyperperistalsis due to increases in serotonin secretion and motilin receptor expression [2]. Ghrelin and motilin concentrations are higher in colicky infants than in controls, supporting the hypothesis that transient dysregulation of the gastrointestinal nervous system during development may contribute to intestinal hypermotility in infants with colic [40].

For several decades, the role of dietary lactose in infantile colic has been an area of debate. Independent of the racial background, term infants generally produce adequate levels of small intestinal lactase to digest around 60 g of lactose daily, corresponding to over 800 mL of breast milk or 1 L of cow’s milk [41]. However, in healthy infants, a small proportion of ingested lactose reaches the colon. Colonic fermentation of lactose causes intestinal hydrogen production, which has been demonstrated to be increased in a high proportion of infants with colic [42,43]. The increased fermentation has been suggested as a factor in the onset of infant colic [44,45].

Different patterns of gut microbiota composition in colicky and non-colicky infants have been demonstrated [46]. Infants with colic commonly have low abundances of *Bifidobacteria* and *Lactobacillus* spp., and an increase in anaerobic Gram-negative bacteria. The high abundance of *Enterobacteriaceae*, especially *Escherichia coli*, may indicate that coliform fermentation in the gut may contribute to the excessive gas production and pain observed in infants with colic [45]. Gut dysbiosis has also been associated with an increased inflammatory pattern and altered immune regulation in colicky babies [47]. The reasons for colic usually disappearing around 4 to 5 months of age cannot be fully explained by changes in microbiota.

## 5. Colic and Long-Term Outcomes

As infant colic usually resolves around 4 to 5 months of age without specific treatments, colic is considered a benign condition. However, colic places a significant burden on the parents and family [2,48]. While the vast majority of infants with colic recover uneventfully, some may be at risk for the later development of behavioral problems, atopy/allergy [49] or feeding difficulties [50]. Many studies display an association between infant colic and the onset of DGBI later in life, probably related to the presence of common pathogenic factors (environmental, dietary, dysbiosis, intestinal dysmotility and visceral hypersensitivity) [51,52]. Castro-Rodriguez et al. collected data in a large, prospective study of 983 infants enrolled at the 2-month well-infant clinic [53]. In that cohort, physician-reported infantile colic occurred in 9.2% (90/983). Markers of atopy (total serum IgE and allergy skin prick test), allergic rhinitis, asthma, wheezing and peak flow variability were the main outcome measures studied at different ages between infancy and 11 years. Children who received a diagnosis of colic by a pediatrician during their first 2 months of life did not have an increased incidence of asthma or wheeze at preschool and school ages compared with children without a history of infant colic [53]. However, Savino et al. [54] reported different results in a prospective study of 96 infants (48 with colic and 48 controls) who were reassessed at the age of 10 years [54]. In that study, allergic rhinitis and conjunctivitis were found in 27% of colicky infants versus 4% of controls (*p* < 0.05; RR 5.4); atopic eczema in 31% of colicky infants versus 6% of the controls (*p* < 0.05; RR 6.3); and food allergy in 22.9% of colicky infants and in 6% of the controls (*p* < 0.05; RR 4.0) [54]. In a recent prospective study in 1249 children, Switkowski et al. reported that the risk of 2–3 concurrent atopic conditions (i.e., eczema, allergic rhinitis and/or asthma) was nearly twice as high in children with a history of colic, compared to those without [55].

Although allergic disease in first-degree relatives has been found to be associated with an increased risk of infant colic, the role of infant colic as a predictor or risk factor for developing later allergic disease is controversial [49,51].

At school age, children with a history of hospitalization for persistent crying in infancy had a significantly higher prevalence of mental health problems and mental disorders, as compared with community samples [56].

## 6. Diagnosis of CMA

CMA is characterized by a wide spectrum of clinical signs and symptoms due to the immune-mediated responses to milk proteins that can be IgE-mediated, non-IgE-mediated or mixed [14]. The exact prevalence of CMA is confounded by the aspecificity of the symptoms and the lack of precise diagnostic criteria [14]. Therefore, there is no evidence for a difference in prevalence according to racial background, although a higher prevalence has been suggested in Asian and Black populations in comparison to White infants. Typically, in the case of IgE-mediated allergy, symptoms present within 1 h of ingesting cow’s milk or its derivatives (Table 2). Infants presenting different manifestations of non-IgE-mediated CMA, such as allergic proctocolitis, food protein-induced entercolitis syndrome and eosinophilic enteritis are almost always very distressed and respond to the definition of colic regarding the duration of crying [14]. However, because of the presence of other symptoms as well in these infants, the crying in these infants does not fulfill the Rome requirements of colic [14].

For an in-depth discussion about IgE- versus non-IgE-mediated CMA, and CMA in breastfed versus formula-fed infants, we refer to the ESPGHAN position paper and the WAO recommendations [14,59]. Increased specific IgE (sIgE) levels (for milk, alpha-lactalbumin, beta-lactoglobulin or casein) and/or a positive skin prick test for milk are useful indicators of IgE-mediated CMA. However, false-positive/-negative results do occur; hence, guidelines recommend an accompanying allergy-focused history and a sequence of dietary elimination and oral food challenge (OFC) to confirm or refute a diagnosis of IgE-mediated CMA [14,60,61].

The diagnosis of non-IgE-mediated CMA is significantly more challenging since, in clinical practice, there is no reliable and validated laboratory test [62,63]. The diagnosis therefore relies mainly on typical clinical symptoms, with gastrointestinal symptoms being a common presentation of non-IgE-mediated CMA (Table 2). As such, reintroduction of cow’s milk protein after a period of elimination is the best method for diagnosing a non-IgE-mediated CMA. As symptom onset may take several days to occur (except for food protein-induced enterocolitis syndrome (FPIES)), parents should be asked to also document any late-onset reactions [1]. Unfortunately, some parents are reluctant to perform a milk reintroduction due to the fear of a relapse of symptoms. In a clinical trial performed in Belgium where both parents were required to sign an informed consent to proceed with an OFC, 23% of the parents refused the OFC [64]. Tools have been developed to increase awareness of non-IgE-mediated CMA, but these are not diagnostic and should not be used without the involvement of HCPs [65]. One such tool is the Cow’s Milk-related Symptom Score (CoMiSS^TM^) [65,66]. CoMiSS is an awareness tool and not a stand-alone diagnostic tool. A positive score of ≥10 indicates the possibility that CMA might be the cause of the symptoms but still requires an elimination diet followed by reintroduction to confirm the suspicion of a CMA. A score of ≥10 can only be reached in the presence of at least two different symptoms with a high score. Since CoMiSS is a tool to be used by the HCP, scores are based on retrospective recall of symptoms by the parents and physical examination by the HCP. A parent-administered tool, the Pre-CoMiSS, has recently been developed, aiming to collect information prospectively and attempting to provide parents with reassurance and guidance by focusing on normal infant development, but also highlighting when further support from an HCP is required [67].

## 7. Colic and CMA

Since the prevalence of colic is estimated to be ~20% and that of CMA at 2 to 4%, some infants will present with both conditions. Symptoms of CMA frequently appear during the first months of life, often within days to weeks after the introduction of a cow’s milk-based formula into the diet or, rarely, while exclusively breastfeeding [62]. Infants with CMA may present with skin manifestations (20–25%) [68], blood-streaked stools (~6%) [69], frequent regurgitation or vomiting (~30%) [70], and nasal symptoms resembling viral infections unrelated to CMA (~30%) [71]. Due to the high prevalence of DGBI in infancy (25–50%) and the often-subtle presentation of non-IgE-mediated CMA, clinicians frequently consider CMA in the differential diagnosis of colic [11,14]. Diagnosis of non-IgE-mediated CMA remains a challenge because of the absence of a reliable diagnostic test. Difficulties in diagnosing non-IgE-mediated CMA are illustrated in the EuroPrevall studies, since the prevalence of non-IgE CMA was 0% in five countries [10]. There is an overlap of symptoms between colic and CMA, which includes abdominal pain and painful flatus, which often is visible in back arching and the pulling of legs to the belly whilst crying (Table 2). Excessive crying in combination with troublesome regurgitation, vomiting and diarrhea as well as constipation might be indicative for possible CMA. Therefore, CMA is often considered as a possible diagnosis when infants present with these symptoms [33,34]. The persistency of colic beyond 5–6 months of age can also be regarded as a possible distinct factor in an allergy-focused history.

The potential pathophysiological overlap between CMA and colic is suggested by the occurrence of dysmotility, visceral hypersensitivity and dysbiosis in both conditions, as well as the beneficial effect of dietary changes [14,72]. However, various study designs, inadequate identification of atopy in participants and differing dietary approaches make it difficult to fully understand the role of CMA in infant colic.

The link between CMA and colic has long been considered, but studies were often methodologically flawed [73]. Reports from as early as the 1950s suggested colic as a manifestation of CMA, but these findings are confounded by incomplete datasets, poorly defined diagnostic criteria, retrospective design and a lack of controlled food challenges. Forsyth noted that excessive crying was a common reason for formula changes, with 26% of mothers suspecting CMA [8]. Yet, concern was also raised about inappropriate formula switches leading to misattributed “illness” in infants [74].

Early studies also proposed a link between maternal cow‘s milk intake and colic in breastfed infants, though such conclusions were prone to observer bias and lacked rigorous controls [21].

Conversely, several studies have reported no significant relationship between CMA and colic. Liebman and Minford et al. found that colic was rarely the sole manifestation of CMA [75,76]. Larger cohort data also suggest similar colic prevalence among breastfed, formula-fed and mixed-fed infants, challenging the dietary protein hypersensitivity hypothesis [77].

Therefore, while CMA may contribute to colic in a subset of infants, particularly those with additional allergic features, colic alone is not a definitive indicator of CMA, whether IgE- or non-IgE-mediated. In fact, HCPs need to be aware of the possibility of over-diagnosis of CMA, as shown by Munblit et al., and not only rely on single symptoms as outlined in CMA guidelines [78]. An allergy-focused history and clinical judgment, supported by a 2–4-week CM elimination diet followed by reintroduction or OFC, remains a key finding for diagnosis and management. Skin prick testing or sIgE allergy testing is generally not indicated in infants with colic, unless an IgE-mediated CMA is suspected. Although allergic disease in first-degree relatives has been found to be associated with an increased risk of infant colic, the role of infant colic as a predictor or risk factor for developing later allergic disease remains controversial [49,51,53].

## 8. Discussion

The Discussion section will focus on the dietary management of infant colic.

While further data on the relationship between both conditions are awaited, if CMA is suspected in a baby with persistent colic (i.e., a family history of atopy or other CMA symptoms) not responding to parental reassurance and education, a 2–4-week trial of a hypoallergenic formula, or a maternal hypoallergenic diet for breastfed infants, may be considered [14,79].

### 8.1. Lactase Supplementation or Lactose-Free Formula

Several randomized studies examining the effects of lactase supplementation in infants with colic have reported a reduction in crying duration, although a recent systematic review was inconclusive due to a lack of standardized clinical definitions and outcome criteria used [80]. That review evaluated the efficacy and safety of lactase supplementation for colic in five randomized controlled trials with 391 infants under six months compared to placebo or no intervention [80]. Three reported reduced crying duration, but only one showed a significant effect in a compliant group (40.4%, *p* = 0.005). However, a meta-analysis of two randomized controlled trials (RCTs) found no significant difference in crying and fussing time compared to placebo [81]. The risk of bias varied among these studies. Similarly, feeding a lactose-reduced formula has been shown to reduce exhaled hydrogen concentrations in infants with colic, but the associated effects on colic symptoms were generally poorly documented [82]. In view of the existing clinical data and negative systematic reviews, lactase supplementation or use of lactose-reduced or lactose-free formula can at this stage not generally be recommended in infants with excessive crying. However, a limited trial of lactase supplements in breastfed infants, or lactose-restricted formula, may be considered on a case-by-case basis in infants with persistent colic symptoms, particularly when non-responsive to general measures.

In particular, in infants with abdominal bloating, flatulence, loose stools or a perianal rash with excoriation due to acidic stools, lactose malabsorption due to small intestinal lactase deficiency should be suspected. In these infants, a range of possible diagnoses associated with secondary lactose malabsorption should be considered, including viral gastrointestinal infection (e.g., rotavirus or norovirus) or non-IgE-mediated CMA with an enteropathy and small intestinal damage [42,83]. The dietary management in infants with manifestations of lactose malabsorption depends on the underlying condition. However, there is no clear evidence that lactase supplementation significantly improves crying in unselected colicky infants [81,84].

### 8.2. Probiotics

There is a growing body of research supporting the treatment of colic with probiotics. The strongest evidence to date exists for *Limosilactobacillus* (*L.*) *reuteri* DSM 17938 and *Bifidobacterium* (*B.*) *animalis* subsp. lactis BB-12 for the treatment of infantile colic in breastfed infants [85,86,87]. However, there is some supportive data for other probiotics, which includes a study by Tyrsin et al. using a different strain of *L. reuteri* (NCIMB 30351) where infants were observed for 25 days and found to have a significant reduction in the number of episodes of colic [88]. Furthermore, a randomized, double-blind, placebo-controlled trial for infantile colic involving supplementation with *Lacticaseibacillus rhamnosus* GG (LGG) for 28 days in breastfed infants, in addition to a crying time reduction, observed a significant decrease in TNF-α in colicky infants treated with LGG, compared to the placebo group [89]. Another study assessing a synbiotic mixture (*L. rhamnosus* PHA-113; *Lactobacillus acidophilus* PHA-121, *B. infantis* PHA-211; *B. lactis* PHA-222; *Streptococcus thermophilus* PHA-311; fructo-oligosaccharides) reported a reduction in colic symptoms in both breastfed and formula-fed infants [90].

According to a recent meta-analysis and systematic review, probiotics resulted in an average reduction of 51 min of crying per day (*p* = 0.001). Further analysis of subgroups showed that the reduction was −39.30 min for vaginal delivery (*p* = 0.003), −64.66 min for *L. reuteri* DSM 17938 (*p* = 0.03), −40.45 min for other strains (*p* < 0.00001), −74.28 min for exclusively breastfed infants (*p* = 0.0003) and −48.04 min for mixed feeding (*p* < 0.00001) [87]. All probiotic strains assessed in this systematic review seemed effective in treating infantile colic. Additionally, it was found that probiotic supplementation was associated with a greater reduction in crying time in exclusively breastfed infants, compared to formula-fed infants. It is not clear why some specific strains of probiotics are more effective in exclusively breastfed infants [87].

The available evidence on the effectiveness of probiotics in formula-fed and cesarean section-delivered infants is scant and needs further research [87,88,91,92]. High placebo response rates (~70%) have been reported in one trial with probiotics [93].

### 8.3. Cow’s Milk-Free Diet

The main findings of 18 studies focusing on the effect of a cow’s milk elimination diet in colicky infants are reported in Table 1. In the early studies, mostly including small populations (ranging from 17 to 66 breastfed or formula-fed colicky infants), the diet intervention showed conflicting results (from no significant reduction in crying to 89% of infants who improved).

In 1991, an Italian group reported that 50/70 (71.4%) formula-fed infants presenting with colic had a remission of symptoms when cow’s milk protein was eliminated from the diet and was replaced by soy formula, and all 50 had two positive oral food challenges [93]. In the patient description section, the included infants also presented with regurgitation, flatulence and poor sleep [20]. Another Italian study showed no difference between a diet without cow’s milk protein, eggs or fish versus dicyclomine in breastfed infants, or soy formula (65.9% versus 53.3%) in formula-fed infants [28]. However, 95.4% of formula-fed infants who did not respond to the initial treatment improved with an extensive casein-hydrolyzed formula [28]. Nevertheless, as infants get older, this subsequent improvement may have been related to the natural evolution of colic.

A double-blind Australian study analyzed the change in the distress ratio after one week of cow milk elimination or control diet in 38 bottle-fed and 77 breastfed infants with colic. After adjusting for age and feeding mode, the authors found that infants on a low-allergen diet experienced a 39% reduction in distress (95% confidence interval (CI), 26–50) compared to a 16% reduction (95% CI, 0–30) for those on the control diet (*p* = 0.012) [94]. However, no separate analysis for breast- or formula-fed infants was performed in this cohort and, obviously, the intervention in the breastfed group was “open”. In another study, the same authors reported that dietary modification, particularly that of breastfeeding mothers whose infants present with colic before the age of 6 weeks, alleviated symptoms [35]. This Australian team also identified a group of infants with distressed behavior attributed to gastroesophageal reflux who failed to respond to histamine 2-receptor antagonists, prokinetic agents and multiple formula changes [35]. Symptoms resolved on commencement of an elemental amino acid-based formula in non-breastfed infants. In two-thirds of the patients, symptoms relapsed when challenged with soy formula or extensively hydrolyzed cow milk formula [35].

According to the systematic review by Lucassen et al. from 1998, which analyzed the outcomes of 27 controlled trials with different interventions, elimination of cow’s milk protein was effective when substituted by hypoallergenic formula (effect size 0.22 [95% CI 0.09 to 0.34]) [82]. The effectiveness of substitution with soy-based formula was unclear when only trials of good methodological quality were considered [82]. The advice to reduce stimulation was beneficial (effect size 0.48 (0.23 to 0.74)), whereas the advice to increase carrying and holding seemed not to reduce crying [82]. The conclusion of this review was that infantile colic should preferably be treated by advising caregivers to reduce stimulation and with a one-week trial of a casein- or whey-based extensive hydrolysate [82]. This recommendation was supported by one double-blind randomized placebo-controlled trial performed by the same authors of the review [26]. In a small group of 15 infants (mean age 5 weeks), a one-week open trial with an extensive casein hydrolysate resulted in significant improvement of colic [22]. Another open trial in only six infants showed a reduction in crying within 1–2 days when fed an amino acid formula [22]. After improvement, infants were challenged with oral doses of 75 mg of bovine immunoglobulins at a 1 mg/mL concentration, which resulted in a relapse of crying and fussiness [16].

In 2005, Hill et al. published an RCT in 107 breastfed infants with severe distress (mean cry/fuss duration >630 min per 48 h) and compared the effect of a low-allergen maternal diet versus a control diet for 7 days. In the low-allergen group, there were significantly more responders (74% vs. 37% reduced cry/fuss duration ≥ 25% from baseline) (95% CI 18–56%) and a more significant cry/fuss duration reduction per 48 h (adjusted geometric mean ratio 0.79, 95% CI 0.63–0.97), with an average reduction of 21% (95% confidence interval: 3–37%) [19].

By contrast, a maternal cow’s milk elimination diet did not significantly reduce crying time in 111 breastfed colicky infants when compared to a control group, although it led to an improvement in three infants (2.6% of the total cases) with positive skin tests [27].

In a systematic review on the dietary management of colic published in 2012, Iacocou et al. concluded that, in formula-fed infants, colic may improve after changing from a standard cow milk formula to either a hydrolyzed protein formula or a soy-based formula whilst fiber-supplemented formulas had no effect [95].

The 2018 Cochrane review of dietary modifications for the treatment of colic found that data were insufficient and at significant risk of bias [96]. The low number of available studies had small sample sizes, and most had serious methodological limitations. In many studies, dietary changes were not limited to hydrolyzed protein formulas but also eliminated lactose, as these formulas were also lactose-free. Benefits reported for hydrolyzed formulas were considered inconsistent [96]. Noteworthily, in this Cochrane review, infant colic was still defined as “full force crying for at least three hours per day, on at least three days per week, for at least three weeks” [96], in line with Wessel’s old criteria.

The recent position paper on CMA of the European Society of Pediatric Gastroenterology, Hepatology and Nutrition clearly states that there are insufficient data to recommend a cow’s milk elimination diet for infants who present with crying and irritability as their sole symptoms [14]. However, when treating infant colic that meets the Rome IV criteria and where CMA is suspected based on additional symptoms, a time-limited elimination diet for 2–4 weeks can be trialed, followed by an OFC or reintroduction [14].

Although, currently, no data are available on rice hydrolysate infant formulas in infant colic, these can be considered as a valuable alternative option to cow’s milk-based extensive hydrolysates, since recent guidelines on the management of CMA position both options as a “first choice option” in the management of CMA [14,57].

## 9. Practical Tips and Conclusions

The description and perception of infant colic depend entirely on the caregivers’ ability to tolerate the crying. The terminology used in describing colic symptoms (e.g., “crying”, “distress”, “irritability” or “fussing”) has overlapping meanings and varies based on language and culture. The diagnosis of infant colic depends on the duration of crying and is therefore dependent on the reliability of parental reporting.

**Figure 2:**
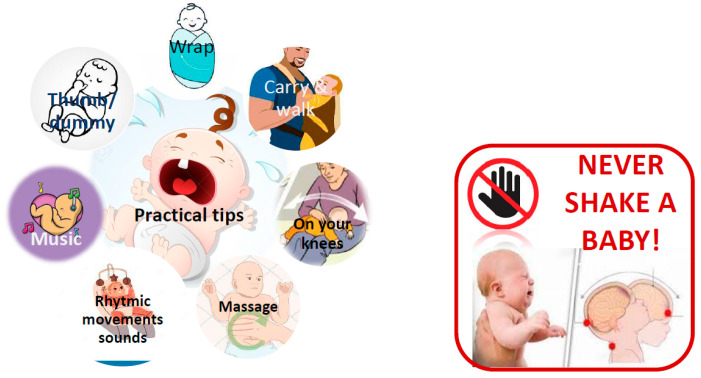
Colic: practical tips.

Excessive crying and unsettled behavior in infancy are, in the vast majority, a manifestation of a DGBI. Since there is no specific treatment for infant colic, general practical tips to sooth a baby are recommended (Table 3 and Figure 2). In a small subset of infants with persistent distress, a specific medical disorder such as GERD or lactose intolerance may be identified. However, empirical treatment with anti-reflux medications, lactase drops or lactose-restricted formula is generally not recommended in infants with crying as the sole clinical presentation of colic.

In the absence of other allergic manifestations, the role of CMA in infant colic remains uncertain [78]. When standard treatment of infant colic is ineffective (Table 1) and if other allergic symptoms are present, CMA may be considered in some cases. When there is a suspicion of CMA, based on the simultaneous presence of persistent crying and other potential allergic symptoms or a positive CoMiSS, a 2–4-week trial of a maternal cow’s milk elimination diet in the breastfed infant, or an extensively hydrolyzed cow’s milk protein formula or hydrolyzed rice formula in formula-fed infants should be considered [14,78]. If the elimination diet is evaluated as efficacious, a timely reintroduction of cow’s milk protein into the diet after the elimination diet is mandatory to confirm the diagnosis of CMA.

Table 4 summarizes and reflects the opinions of the authors on statements regarding the etiology, diagnosis and management of infant colic and its differential diagnoses.

When standard treatment of infant colic is ineffective, and other symptoms possibly indicate CMA, or in the presence of a positive scoring of a validated awareness tool (CoMiSS), CMA may be considered in some cases. If CMA is considered as a possible etiology, the guidelines for the diagnosis and management of CMA should be applied [14]. Because current evidence is still limited, research should focus on large well-designed clinical trials investigating CMA in colicky breastfed and formula-fed infants. RCTs in formula-fed infants with infant colic fulfilling the Rome criteria comparing extensively hydrolyzed cow’s milk-based formula, with and without lactose, versus rice hydrolysates will contribute to finding an answer for the role of lactose vs. CMA in infant colic. Also, RCTs with identical formulas except for the presence of ‘biotics’ (prebiotics, probiotics, synbiotics, postbiotics) evaluating the effect of these biotics on the gastrointestinal microbiota composition and their relation to crying duration would further highlight the relation between microbiota and colic.

## Figures and Tables

**Table 1:** Clinical trials assessing cow’s milk elimination in infantile colic.

Author, Source	Population and Intervention	Diagnosis of CMA/Results of Challenge	Main Results
Campbell [15]	19 FF colicky infants put on soy-F vs. StF (DBRCT) (no IgE testing)	8/11 infants who responded to soy had positive challenge at 3 months, 0/8 at 6 months The 2 infants who responded to eHF relapsed on CMP	In 13/19 infants (88%), colic disappeared after the dietary change: colic disappeared in one week in 11/19 infants put on soy; 2/8 infants who did not respond to soy improved on eHF
Estep [16]	6 colicky infants started AAF for 5–17 days (no IgE testing)	All had challenge with oral doses of 75 mg of bovine IgG at a 1 mg/mL dose and increased crying and fussing	All infants improved, usually within 1–2 d. The total time spent crying and fussing was reduced by an average of 45%, representing a decrease of 1.0 to 5.2 h daily.
Evans [17]	20 BF persistent colicky infants; DBRC cross-over trial, maternal intake of CM or soy milk or CM + SM in blocks of 2 days (no IgE testing)	Indirect series of challenges over a study period of 12 days	Avoidance of CM produced no beneficial effects on the incidence of colic in the babies
Forsyth [18]	17 FF colicky infants put on casein-eHF vs. StF, 3 formulas changes of 4-day periods (DB cross-over study) (no IgE testing)	Repeated challenge during the study period of 16 days	Significantly less crying and colic in infants fed eHF vs. StF; with the second change, only less colic on eHF. No difference by the third formula change
Hill [19]	90/107 BF colicky infants put on low-allergen maternal diet vs. control diet for 7 days (RCT) (no IgE testing)	No challenge	In the low-allergen group, significantly more responders (74% vs. 37%) (absolute risk reduction of 37%, 95% CI 18–56%), greater cry/fuss duration reduction per 48 h (adjusted geometric mean ratio 0.79, 95% CI 0.63–0.97) with an average reduction of 21% (95% confidence interval: 3–37%)
Iacono [20]	70 FF severe colicky infants put on soy-F (no IgE testing)	Two successive challenges caused the return of symptoms in all the 50 responsive infants	In 50/70 (71.4%), colic disappeared on soya-F within 48 h. At the mean 18-month follow-up period, 22/50 (44%) food intolerance vs. 1/20 (5%) of those with non-CMP-related colic
Jakobsson [21]	19 BF colicky infants; 18 mothers put on CMFD (no IgE testing)	12/13 relapses of colic on ≥2 indirect challenges (CMP in mothers’ diet)	Disappearance of colic in 13/18 (72%)
Jakobsson [22]	22 FF colicky infants fed 2 different casein eHFs (no IgE testing)	11/14 subjects showed a positive response to whey (median 2.4 h/d), 10/14 to milk (median 2.1 h/d) and 2/13 to placebo (median 1 h/d)	15/22 significant reduction in colic. No difference between the 2 eHFs
Jakobsson [23]	66 BF colicky infants: mothers put on CMFD (no IgE testing)	23/66 (35%) relapse of colic on ≥2 indirect challenges (CMP in mothers’ diet); among the 23 responders, 9/10 infants reacted with colic when mothers took whey protein-containing capsules (DB cross-over trial)	Disappearance of colic in 35/66 (53%)
Lothe [24]	60 FF colicky infants put on StF or Soy-F (DBRCT); if no response casein-eHF (no IgE testing)	At challenge with StF after 1 month, relapse of colic in 22 infants (36%); at age 6 months, positive challenge in 11 infants (18%); at 12 months in 8 infants (13%) and at 16 months in 5 infants (8%)	In 11/60 (18%) infants, colic disappeared on soya; 32/60 (53%) with persistent colic responded to casein-eHF
Lothe [25]	27 FF infants with severe colic put on casein-eHF (no IgE testing)	Among the 24 responders to eHF, when given whey protein-containing capsules (DB cross-over trial), colic relapsed in 18 infants vs. 2 infants who relapsed with placebo	In 24/27 (89%) infants, colic disappeared on a casein-eHF and crying decreased from 5.6 h/d to 0.7 h/d. Crying was 3.2 h/d for the infants receiving whey protein capsules vs. 1.0 h for those receiving placebo.
Lucassen [26]	43 FF colicky infants put on whey eHF or StF (DBRCT) (no IgE testing)	No data of challenge	A difference in the decrease in crying duration of 63 min/day (95% CI: 1–127 min/day) in favor of the whey eHF
Moravej [27]	114 BF colicky infants (IgE testing: SPT)	In the 3/114 infants with positive SPT, colic disappeared when mothers were on CMFD	Maternal diet did not significantly reduce crying hours in infants with negative SPT
Oggero [28]	120 BF or FF infants with severe colic fed hypoallergenic diet (low-allergen diet with mother or soy milk/eHF in FF) (group A) vs. dicyclomine hydrochloride (group B) (no IgE testing)	No challenge performed	In BF infants, no significant improvement with diet (10/16, 63% vs. 10/15, 66%). In FF, no difference between soy and dicyclomine (29/44, 66% vs. 24/45, 53%); significant improvement in infants on eHF vs. dicyclomine (95.4 vs. 53.3%)
Taubman [29]	21 BF or FF colicky infants; maternal CMFD in BF or casein-eHF in FF vs. parental counseling and normal diet (RCT) (no IgE testing)	No infant who improved with dietary changes had a significant increase in crying, when re-exposed to CMP	In the dietary changes group, the crying decreased from 3.19 ± 0.69 h/d to 2.03 ± 1.07 h/d, less than in the counseling group (from 3.21 ± 1.10 h/d to 1.08 ± 0.70 h/d)
Verwimp [30]	79 infants with diagnosis of CMPI fed with 2 different whey-eHF (no IgE testing)	Diagnosis of CMPI as defined by standard elimination/provocation test in primary healthcare setting	Symptom improvement (as for severity of eczema and infantile colic) reported in 80% of infants

Legend: BF = breast-fed; CI = confidence interval; CMA = cow’s milk allergy; CMFD = cow’s milk-free diet; CMP = cow’s milk protein; CMPI = cow’s milk protein intolerance; DB = double blind; eHF = extensive hydrolyzed formula; FF = formula-fed; h/d = hour/day; min/day = minutes per day; Soy-F = soy-based formula; SPT = skin prick test; StF = standard formula.

**Table 2:** Summary of symptoms considered for non-IgE-mediated allergy according to different guidelines.

	ESPGHAN [14]	DRACMA [57]	iMAP [58]
Gastrointestinal	Food refusal Dysphagia Regurgitation, vomiting Diarrhea Constipation Blood in the stool	Diarrhea Vomiting Colic Hematochezia	Colic (persistent irritability) Vomiting/reflux/GERD Diarrhea Constipation Abdominal discomfort Painful flatus Blood in the stool Mucus in the stool
Skin	Eczema (atopic dermatitis) Perianal rash Anal fissures	Angioedema Urticaria Erythema	Pruritus Non-specific rash Atopic dermatitis
Respiratory	Rhinitis, wheezing Chronic cough	Pharyngeal swelling	Acute rhinitis and or conjunctivitis
Other	Colic, irritability Faltering growth Iron deficiency Anemia	Lethargy and pallor after acute vomiting Iron deficiency	Food refusal Faltering growth

**Table 3:** Practical tips. What can I do to soothe a baby with colic?

The following may be helpful to soothe a baby with colic. Remember, every baby responds differently so you may need to try a variety of techniques before finding the ones that work best for your baby.
Swaddling or wrapping your baby in a thin, large blanket can make them feel more secure as it recreates the feeling of the womb. Ask a healthcare professional to show you how to swaddle your baby so that they can’t wriggle free.
Carry your baby in a sling or front carrier on your chest as you walk around. The body contact and motion are calming. To ease wind, lay your baby tummy-down across your knees while gently rubbing the baby’s back.
Massage your baby. Babies love skin-to-skin contact and studies suggest babies who are regularly massaged cry and fuss less. Ask a healthcare professional for information about local baby massage classes.
Steady, rhythmic movements are soothing. Cradle your baby while rocking in a chair or try a baby swing or a vibrating baby seat.
Recreate the soothing womb environment with soft music, a white noise machine, a fan or a recording of a heartbeat.
Help your baby find their hand, fingers or thumbs to suck on or consider offering a dummy to pacify them.
Never shake a baby to stop his/her from crying as this could cause serious and irreparable lesions.
If you are still struggling to calm your baby or you have any concerns about their health, speak to a healthcare professional for further advice.

**Table 4:** Practice points.

Statement		Votes
1	The origin of colic is multifactorial and therefore health care professionals need to have a holistic approach and take a detailed clinical history that also explores parental stress.	9 (7×)
2	When a health care professional is consulted because of inconsolable crying, the primary approach should be to look for/exclude alarming symptoms and undertake appropriate action if present (Figure 1).	9 (7×)
3	It is crucial to reassure parents of the infant presenting with colic, whether breastfed or formula-fed, that this is a disorder of gut-brain interaction, which typically does not need treatment or changes in the maternal diet or change to a special infant formula.	9 (6×); 7
4	Breastfeeding should be promoted, supported and continued as long as the mother can or is willing to breast feed. Transitioning to infant formula will have no impact on the symptoms and is not recommended.	9 (7×)
5	Reviewing feeding practice and volume of feed according to age and weight of the infant should be assessed prior to commencing an elimination diet	9 (7×)
6	A 2–4 week trial of a probiotic with demonstrated efficacy in randomized controlled trials may be considered in infants with colic only after general measures have been reviewed/implemented by HCP	9 (5×); 8; 6
7	Consider CMA in infants presenting with significant colic and where there is no response to general supportive measures such as parental reassurance/education and/or probiotic.	9 (6×); 6
8	Consider CMA in infants if significant irritability/crying is associated with other symptoms, including vomiting/regurgitation, diarrhoea, eczema.	9 (7×)
9	The use of the Cow’s Milk-related Symptom Score (CoMiSS^TM^) is likely to contribute to more accurately selecting infants for a trial with an elimination diet since a higher score (≥10) necessitates the presence of at least two symptoms linked to CMA.	9 (7×)
10	CoMiSS^TM^ is an awareness tool and not a diagnostic stand-alone tool.	9 (7×)
11	In formula fed infants, soy formula is not indicated for colicky infants [97].	9 (7×)
12	Published evidence on the effects of lactase supplementation or use of lactase-restricted formula in breastfed or formula-fed infants remains inconclusive. Use of lactase drops or lactose restriction in infants with typical colic symptoms is therefore generally not recommended, and further research is needed.	9 (6×); 8
13	When a trial of a maternal cow’s milk elimination diet is deemed appropriate, the healthcare professional needs to ensure dietary adequacy for the mother including vitamin and mineral supplementation.	9 (6×); 8
14	An extensively hydrolyzed formula or hydrolyzed rice formula may be used in formula-fed infants with colic, as first line choice in infants with possible CMA.	9 (7×)
15	After a 2–4-week elimination diet for either the breastfeeding mother or the formula feeding infant, there should be a reintroduction of cow’s milk protein to confirm or refute the diagnosis of CMA.	9 (7×)
16	Adequate follow-up of any infant with colic and clinical assessment of the treatment response is needed, particularly when infants are on an elimination diet.	9 (6×); 8

## Data Availability

No new data.

## Abbreviations

B	*Bifidobacterium*
BF	Breastfed
CI	Confidence interval
CMA	Cow’s milk allergy
CMFD	Cow’s milk-free diet
CMP	Cow’s milk protein
CMPI	Cow’s milk protein intolerance
CoMiSS	Cow’s milk-related symptom score
DB	Double-blind
DGBI	Disorder of gut–brain interaction
EAACI	European Academy of Allergy and Clinical Immunology
eHF	Extensively hydrolyzed formula
FF	Formula-fed
FGID	Functional gastrointestinal disorder
FPIES	Food protein-induced enterocolitis syndrome
GERD	Gastroesophageal reflux disease
HCP	Healthcare professional
h/d	Hours/day
IgE	Immunoglobulin E
L	*Limisolactobacillus*
LGG	*Lacticaseibacillus rhamnosus* GG
min/day	Minutes/day
OFC	Oral food challenge
RCT	Randomized controlled trial
sIGE	Specific IgE
Soy-F	Soy-based formula
SPT	Skin prick test
StF	Standard formula
